# Letter from the Editor in Chief

**DOI:** 10.19102/icrm.2026.17074

**Published:** 2026-07-15

**Authors:** Devi Nair


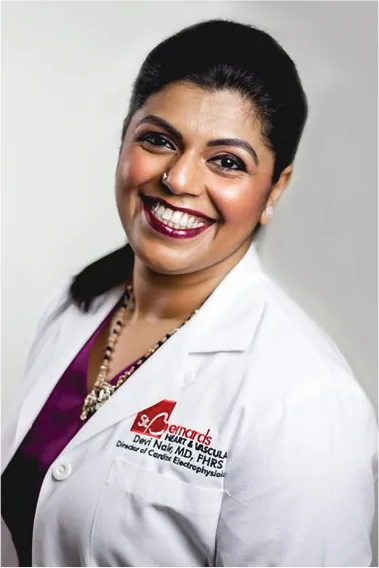


Dear Colleagues,

Welcome to the July 2026 issue of *The Journal of Innovations in Cardiac Rhythm Management*.

This has been a month that reminded me how thoroughly our field has become a global one. In late July, the Japanese Heart Rhythm Society (JHRS) convened its 72^nd^ Annual Scientific Meeting in Kyoto, where I had the privilege of serving as faculty. To stand before those sessions was to watch the world of electrophysiology gather in a single place, with Japanese, European, and North American investigators comparing ablation strategies, mapping technologies, and device therapies and discovering, as we always do at these meetings, how much more unites our practices than divides them. The questions that animate a laboratory in Kyoto are the same ones that fill a laboratory in Kansas City or Ankara or Kolkata, and the answers, increasingly, are arrived at together. International meetings such as the JHRS gathering are not merely occasions for the exchange of data; they are the connective tissue of a worldwide specialty, and I returned from Kyoto renewed in my conviction that the future of our field will be written collaboratively, across borders and time zones.

If the international meetings are where our field takes stock of itself, then the fellows training programs are where it renews itself. I want to use this letter to say a word about the immersive, hands-on educational programs that increasingly bridge the gap between fellowship and independent practice. The Kansas City Heart Rhythm Symposium Fellows Bootcamp and Allied Healthcare Program, an HRS Synchrony Program event scheduled for this August in Overland Park, is a fine example of the form: two days built not around passive lectures but around case-based learning, panel debates, and direct engagement with the tools and techniques that fellows will carry into their careers designed deliberately for the whole electrophysiology team, including both general cardiologists and electrophysiologists and the nurses, nurse practitioners, and laboratory professionals on whom every successful practice depends. I would encourage our trainees and their program directors to seek out this and other bootcamps. There is a kind of learning that happens only when a fellow is put in front of a problem, given the instruments, and asked to reason aloud in the company of peers and mentors, and it is precisely this kind of learning that a busy clinical service, for all its virtues, cannot always provide.

The manuscripts collected in this issue reflect the breadth of what those trainees must eventually master, from the machine-learning classification of atrial wavefronts to the bedside recognition of a metabolic emergency that silences an implanted device. They span two original research contributions, a contemporary review of a fast-moving technology, a cautionary case report, and a tracing that rewards close reading.

In an original research contribution, Castellano, Kong, D’Arcy, and colleagues^1^ analyze 2,933 electrographic flow recordings from 151 patients and train a neural-network classifier that distinguishes atrial fibrillation from the more organized flutters and atrial tachycardias with 81% accuracy and flawlessly separates atrial fibrillation from organized rhythms. It is an instructive glimpse of computational tools that read the atrium as a field of motion.

In a second original contribution, Ozbay, Demirhan, Degirmen, and Ozcan^2^ pool four studies totaling 1,096 patients and find that frailty is associated with a significantly higher risk of recurrence after atrial fibrillation ablation. As our ablation populations grow older, frailty deserves a place in pre-procedural risk stratification.

Ghaleb, Kolawole, and colleagues^3^ review the pulsed field ablation landscape and the platforms cleared by the US Food and Drug Administration to date, tracing the technology from the first single-shot systems toward focal and hybrid designs. For a field that has moved from experimental to front-line in only a few years, it is a clear and useful map.

Roche, Kanan, and Mikhalkova^4^ from Saint Louis University describe a woman with BRASH syndrome whose implantable cardioverter-defibrillator failed to capture at a potassium concentration of 7.8 mEq/L; correcting her hyperkalemia restored capture. It is a pointed reminder that a pacemaker captures only the myocardium the metabolism will allow.

Finally, in this month’s Tracing of the Month, Kara, Ozeke, and colleagues^5^ from Ankara Bilkent City Hospital use a case of recurrent ventricular tachycardia to show that termination after a single extrastimulus does not always signify capture of the critical isthmus. It is a small master class in the careful reading of the response to pacing.

What runs through this issue, and through the meetings I have described, is the same conviction: that our field advances by teaching itself, generously and continuously, across institutions and across borders. The fellow who reasons aloud at a bootcamp in Overland Park, the investigator who presents in Kyoto, and the author who submits a careful case to these pages are all engaged in the same work. I will close, as I often do, with an invitation. If you have a study, a review, a case, or a tracing that taught you something worth sharing, this journal exists to publish it, and we are especially glad to see the work of trainees and of the international community that makes our specialty what it is.

I am grateful, as always, to our authors, reviewers, and editorial team for their work, and to you, our readers, for your continued engagement with the journal.

Warm regards,


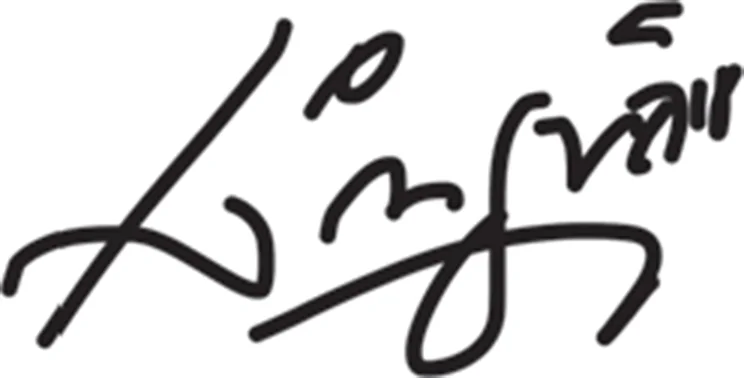


Dr. Devi Nair, md, facc, fhrs

Editor-in-Chief

*The Journal of Innovations in Cardiac Rhythm Management*

Director of the Cardiac Electrophysiology & Research,

St. Bernard’s Heart & Vascular Center, Jonesboro, AR, USA

White River Medical Center, Batesville, AR, USA

President/CEO, Arrhythmia Research Group

Clinical Adjunct Professor, University of Arkansas for Medical Sciences

Governor, Arkansas Chapter of the American College of Cardiology

drdgnair@gmail.com
